# Nucleolin-Targeting AS1411 Aptamer-Conjugated Nanospheres for Targeted Treatment of Glioblastoma

**DOI:** 10.3390/pharmaceutics16040566

**Published:** 2024-04-21

**Authors:** Kyeongjin Seo, Kihwan Hwang, Kyung Mi Nam, Min Ju Kim, Yoon-Kyu Song, Chae-Yong Kim

**Affiliations:** 1 Department of Neurosurgery, Seoul National University Bundang Hospital, Seongnam-si 13620, Republic of Korea; sian61@snu.ac.kr (K.S.); coolghh@gmail.com (K.H.); hyukmn82@gmail.com (K.M.N.); 2 Department of Health Science and Technology, Graduate School of Convergence Science and Technology, Seoul National University, Seoul 08826, Republic of Korea; 3 Department of Neurosurgery, Seoul National University College of Medicine, Seoul 03080, Republic of Korea; 4 Astrogen Inc., 440, Hyeoksin-daero, Dong-gu, Daegu 41072, Republic of Korea; mjkim@astrogen.co.kr; 5 Department of Applied Bioengineering, Graduate School of Convergence Science and Technology, Seoul National University, Seoul 08826, Republic of Korea; 6 Advanced Institutes of Convergence Technology, Suwon-si 16229, Republic of Korea

**Keywords:** glioblastoma, AS1411, nucleolin, DNA nanostructures, targeted drug delivery

## Abstract

Post-operative chemotherapy is still required for the treatment of glioblastoma (GBM), for which nanocarrier-based drug delivery has been identified as one of the most effective methods. However, the blood-brain barrier (BBB) and non-specific delivery to non-tumor tissues can significantly limit drug accumulation in tumor tissues and cause damage to nearby normal tissues. This study describes a targeted cancer therapy approach that uses AS1411 aptamer-conjugated nanospheres (100–300 nm in size) loaded with doxorubicin (Dox) to selectively identify tumor cells overexpressing nucleolin (NCL) proteins. The study demonstrates that the active target model, which employs aptamer-mediated drug delivery, is more effective than non-specific enhanced permeability and maintenance (EPR)-mediated delivery and passive drug delivery in improving drug penetration and maintenance in tumor cells. Additionally, the study reveals the potential for anti-cancer effects through 3D spheroidal and in vivo GBM xenograft models. The DNA-protein hybrid nanospheres utilized in this study offer numerous benefits, such as efficient synthesis, structural stability, high drug loading, dye labeling, biocompatibility, and biodegradability. When combined with nanospheres, the 1411 aptamer has been shown to be an effective drug delivery carrier allowing for the precise targeting of tumors. This combination has the potential to produce anti-tumor effects in the active targeted therapy of GBM.

## 1. Introduction

GBMs are the most frequent brain tumors and are characterized by a very poor prognosis. Typical survival after diagnosis is approximately 14.6 months, and only 20–25% of patients show improvement in 2-year survival with approved treatments [1,2]. Attempts to extend the median survival with central nervous system (CNS) drugs have been limited by the complexity of the brain, particularly the difficulty of penetrating the blood-brain barrier (BBB) [1,3]. The BBB is a major barrier to drug delivery in most brain tumors [4,5,6,7]. As the tumor progresses, the BBB is referred to as the blood-tumor barrier (BTB), which differs from the BBB in that it is damaged and new blood vessels are formed. In GBM, the permeability of the BTB is high in the bulk of the tumor, but there is very little permeability in the periphery [2]. Accordingly, a GBM cell-specific target-binding strategy may be required for drug delivery across the BTB.

Chemotherapeutic drugs, such as temozolomide (TMZ), paclitaxel (PTX), and doxorubicin (Dox), are used for the treatment of glioblastoma, but these drugs have limitations such as low solubility, stability, and nonpenetration [8,9]. Nanoparticles are a promising solution due to the improvement of drug delivery efficiency and a reduction in side effects [10,11,12]. Dox exerts anti-tumor activity through inhibiting DNA replication, transcription, and repair through intercalation into DNA base pairs, blocking these processes [13,14]. As such, Dox was selected as a drug due to its ability to act as a carrier and insert into the DNA strand. Nanopharmaceutical research has been continuously reported in cancer treatment applications, and numerous nanocarrier-based drug delivery systems have been considered, including the development of specific tumor-targeted drug delivery systems [15,16,17,18,19,20]. In addition, nanocarrier designs using biomolecules, such as DNA, aptamers, and proteins, have been reported to minimize side effects [21,22,23]. Among the nanocarrier synthesis studies using biomaterials, it is highly efficient to induce self-assembled hybrid systems through biotin–streptavidin hydrogen-bonding interactions and complementary base pairs of DNA [24,25,26,27,28]. DNA has excellent cell permeability and relative resistance to enzymatic degradation, and the streptavidin–biotin interaction has the advantages of high specific molecular recognition and resistance to changes in temperature or pH, which can form a stable structure [28,29,30,31]. Nanoparticle assembly techniques provide a method for self-assembly through exploiting the strong binding affinity of the biotin–streptavidin interaction. Streptavidin, in combination with biotinylated DNA, can induce self-assembly as a cross-linker, allowing for the construction of a structure in a nanoparticle drug delivery system using nucleic acids [32,33,34]. Here, we apply previous research techniques to synthesize nanocarriers using only biopolymers that induce biotin–streptavidin molecular binding based on DNA structures. The objective of this innovation is to fabricate hybrid nanocarriers by employing the probabilistic pairing of streptavidin (STV) and double-stranded DNA (dsDNA) long chains [35]. STV, existing as homotetramers, exhibits the capacity to bind with up to four biotin units [36]. For DNA and aptamers, transformations yielding biotin-binding modified structures are generated and utilized.

Nanodrug carriers optimize pharmacokinetics by leveraging the EPR effect for tumor accumulation while maintaining a negative zeta (ζ) potential [37,38]. An effective nanocarrier design encompasses considerations of size and ζ potential. Building upon our previous work, we utilized a hybrid nanosphere composed of DNA and streptavidin in this study, anticipating enhanced suspension stability with a near-zero ζ potential [35]. Consistent with our prior findings, we expected the incorporation of streptavidin into dsDNA to align the nanodimer′s ζ potential with streptavidin, and the inclusion of aptamer and doxorubicin (Dox) to further decrease the ζ potential, facilitating tumor targeting.

An aptamer is a DNA or RNA oligonucleotide that can recognize a specific three-dimensional structure, even with a short base sequence, and can bind to a target protein [39,40,41]. In addition, the aptamer is used as a suitable candidate for target delivery due to its low molecular weight, small size, non-immunogenicity, inherent biocompatibility, and stable structure in biological environments [42,43,44]. In particular, AS1411 is a guanine-rich DNA oligonucleotide, which is one of the aptamers reported to be fully effective as an anti-cancer drug in phase 1/2 of clinical trials [45,46,47]. AS1411, an oligodeoxynucleotide aptamer, has shown promise in clinical trials by interfering with NCL, a protein that is overexpressed in GBM [23,48,49,50]. AS1411-decorated nanoparticles have demonstrated enhanced drug delivery to GBM cells, leading to reduced tumor growth and increased survival in animal models [51]. AS1411 recognizes NCL proteins expressed in tumor cell membranes through the high affinity of guanine domains, and the internalization of AS1411–NCL complexes results in the significant inhibition of DNA synthesis, the destabilization of bcl2 mRNA, and the induction of cell apoptosis [23,52,53,54]. NCL is expressed on the cell surface and inside the cell, is overexpressed in highly proliferating types of various cancer cells (including GBM cell lines), and mediates the binding of AS1411 and intracellular uptake [55,56,57,58]. Therefore, the main target for improving GBM treatment in this study was the AS1411–NCL recognition and interactive drug delivery system. Several types of nanoparticles have been used for drug delivery using AS1411–NCL recognition, including poly (D,L-lactic-co-glycolic acid) (PLGA) nanoparticles, docetaxel (DTX)-loaded poly (ethyleneglycol)–poly(ε-caprolactone) (PEG)–(PCL) nanoparticles, and poly (L-γ-glutamyl-glutamine) (PGG)–PTX nanoparticles, for targeted glioblastoma therapy [59,60,61,62]. In this study, we utilize the AS1411 aptamer as a targeted therapy for glioblastoma, similar to previous nanopharmaceuticals. The difference is that efficient drug-loading synthesis is possible through complementary DNA binding and self-assembly synthesis through the streptavidin–biotin interaction, and the resulting product has biocompatibility and can be degraded in vivo.

The aim of this study is to target brain tumor tissue with chemotherapy. A complete surgical resection of brain tumor tissue in GBM patients is challenging, and the prognosis is often poor even after treatment. This study aims to demonstrate the potential of nanomedicine, which has a high target specificity and biocompatibility, for anti-tumor efficacy using biomaterials.

## 2. Materials and Methods

### 2.1. Cell Lines and Cell Culture

The cell lines (human GBM cells; U87 and U251) used in this study were obtained from the Korean Cell Line Bank, Seoul, Korea. The normal human astrocytes (NHAs) cell line was purchased from iXCells Biotechnologies, San Diego, CA, USA. These cell lines were grown and maintained in culture with Dulbecco′s modified Eagle′s medium (DMEM; Biowest, Nuaillé, France) supplemented with 10% fetal bovine serum (FBS; Biowest, Nuaillé, France) and 1% penicillin–streptomycin. The BBB hCMEC/D3 cell line and media were purchased from Millipore, Temecula, CA, USA, and cultured in EndoGRO™-MV Complete Media Kit (Cat. No. SCME004) supplemented with 1 ng/mL FGF-2 (Cat. No. GF003). All cells were maintained at 37 °C in a 5% CO_2_ atmosphere.

### 2.2. Quantitative Real-Time RT-PCR

Total RNA was extracted from cultured cells using Trizol reagent (Invitrogen, CA, USA) according to the manufacturer′s instructions. After DNase treatment with RQ1 RNase-free DNase (Promega, Mannheim, Germany), 1 µg of total RNA was used for first-strand cDNA synthesis with oligo-dT primers using the ProtoScript M-MuLV Taq RT-PCR system (New England Biolabs, MA, USA).

Q-PCR amplification was performed in a Quantstudio 7 real-time PCR system (Applied Biosystems, CA, USA) using 96-well plates. The reaction was performed with a final volume of 25 µL using ABSOLUTE′TM QPCR SYBR Absolute QPCR Green Rox Mix (Thermo Scientific, Vilnius, Lithuania) according to the manufacturer′s instructions. The qPCR conditions were as follows: an initial denaturation at 95 °C for 10 min, followed by 40 cycles with denaturation at 95 °C for 5 s, and the annealing/elongation at 60 °C for 30 s, followed by a dissociation phase at 95 °C for 15 s, 60 °C for 15 s, and 95 °C for 15 s. The primer sequences are as follows: Hu NCL (5′-AGGAGGAGGAAGAAGAGGAG-3′ and 5′-ACAAAGAGATTGAAAGCCGTAG-3′; product size 148 bp); Hu GAPDH (5′-GCACCGTCAAGGCTGAGAA-3′ and 5′-AGGGATCTCGCTCCTGGAA-3′; product size 75 bp). The primer sequences and conditions used in the qPCR to investigate the expression of NCL mRNA were as previously reported [63].

### 2.3. Western Blot Analysis

The cells were washed with ice-cold PBS and lysed on ice with RIPA buffer (Thermo Scientific, Rockford, IL, USA) containing Halt protease and phosphatase inhibitor cocktail (Thermo Scientific). The protein concentration of the supernatant was measured using the Pierce BCA (bicinchoninic acids) protein assay kit (Thermo Scientific), and the lysates were resolved on a 4–20% Mini-PROTEAN TGX precast gel (Bio-Rad Laboratories, CA, USA). The samples were then transferred to 0.45 µm methanol-activated polyvinylidene fluoride (PVDF) membranes (Thermo Scientific) and the membranes were blocked with 5% skim milk solution. The membranes were washed three times with TBS-T, followed by primary antibody incubation overnight. The primary antibodies used were rabbit anti-NCL (D4C7O) monoclonal antibody (Cell Signaling Technology, MA, USA) and rabbit anti-beta actin (13E5) monoclonal antibody (Cell Signaling Technology). The next day, the membranes were washed three times each with TBS-T and incubated with the anti-rabbit IgG HRP-conjugated secondary antibody (Cell Signaling Technology). HRP antibodies were detected using the SuperSignal West Pico PLUS chemiluminescent substrate (Thermo Scientific).

### 2.4. Synthesis of AS1411 Aptamer-Conjugated Nanospheres

#### 2.4.1. Nanoparticle Synthetic Materials and Instruments

Thermo Fisher Scientific Inc. (Invitrogen, Carlsbad, CA, USA) provided the Streptavidin (STV), while Bioneer Co (Bioneer, Daejeon, Republic of Korea) synthesized and purified the biotinylated single-stranded DNA (ssDNA) using high-performance liquid chromatography (HPLC). The biotinylated ssDNA sequences were as follows: 5′-ACGGCTGCGCGACGTAGGTACGGCAACTCGCGGCTATGCA-3′ and 5′-BiotinTEG-TACCTACGTCGCGCAGCCGTTGCATAGCCGCGAGTTGCCG-3′. Each nanosphere sample was prepared by diluting to a final concentration of particles between 10^6^ and 10^9^ in a 1 mL volume of PBS, and the size distribution was measured by injecting it into NanoSight NS300 (Malvern, Worcestershire, UK) with a syringe. Confocal imaging of the FAM-labeled nanospheres was performed using a Zeiss LSM710 confocal laser-scanning microscope (Carl Zeiss, Jena, Germany).

#### 2.4.2. Synthesis of DNAProtein Hybrid Nanospheres Using Self-Assembly

An equimolar mixture of the complementary strands (5′-ACGGCTGCGCGACGTAGGTACGGCAACTCGCGGCTATGCA-3′ and 5′-BiotinTEG-TACCTACGTCGCGCAGCCGTTGCATAGCCGCGAGTTGCCG-3′) was dissolved in PBS to a final concentration of 50 µM. The solution was heated in a water bath at 95 °C for 5 min and slowly cooled to room temperature. Meanwhile, Dox-loaded nanospheres were synthesized by inducing DNA intercalation at a ratio of 1:10 between dsDNA and Dox in a mixture of complementary strands. After the formation of a linear DNA chain, STV and an equivalent amount of DNA monomer were mixed, stored at 4 °C for 3 h, and then used. The complementary strand of sequences and synthesis conditions were performed as previously reported [35,64]. FAM-labeled nanospheres were used as follows: 5′-FAM-ACGGCTGCGCGACGTAGGTACGGCAACTCGCGGCTATGCA-3′.

#### 2.4.3. Conjugation of the AS1411 Aptamer to Nanospheres

In order to construct aptamer-conjugated nanospheres, biotinylated AS1411 aptamer (5′-BiotinTEG-GGTGGTGGTGGTTGTGGTGGTGGTGG-3′) was added and incubated for 10 min at room temperature. Prior to use, the AS1411 aptamer was dissolved in nuclease-free water, heated to 85 °C for 2 min, and cooled to room temperature for 10 min [65]. After completing nanoparticle synthesis, agarose gel electrophoresis and flow cytometric analysis were performed to confirm the absence of ssDNA and aptamer residues.

### 2.5. Agarose Gel Electrophoresis

A 3% agarose gel was prepared in 1 × TBE buffer (890 mM Tris-borate, 890 mM boric acid, 20 mM EDTA, pH 8.0) and stained with SYBR Safe DNA gel stain (Invitrogen, CA, USA). The sample solution (10 µL) was mixed with 2 µL of 6 × loading buffer. The prepared samples were then subjected to electrophoresis (Mupid-exU) in 1 × TBE buffer at 100 V for 30 min. The gel was imaged using the Gel Doc EZ imaging system (Bio-Rad, Hercules, CA, USA).

### 2.6. Characterization of Drug Loading and Release

In order to quantify drug loading on the nanospheres, dsDNA was synthesized with Dox at various concentration ratios (dsDNA:Dox = 1:2, 1:5, 1:10, 1:20, 1:50, 1:100). The fluorescence spectra were collected in the wavelength range of 350–700 nm, with both absorbance and emission slit widths of 10 nm, using an Epoch-2 microplate spectrophotometer (BioTek Instruments, Winooski, VT, USA). In order to study Dox release under the influence of nuclease degradation, 10 µM Dox-Apt-Nanosphere treated with DNAse I (1 U/mL; more than twice the level in human serum) [66,67] was transferred to a dialysis tube (Pur-A-Lyzer™ Midi Dialysis Kit, Sigma-Aldrich, CA, USA) and immersed in PBS (pH 7.4) at 37 °C. The fluorescence of Dox (Ex/Em: 470/560) in PBS was measured, and the amount of drug released was calculated at different time intervals (1, 3, 6, 24, and 48 h) using an Epoch-2 microplate spectrophotometer (BioTek Instruments).

### 2.7. Cytotoxicity Assay

The cells (5 × 10^3^ cells per well) were seeded in 100 µL DMEM medium supplemented with 10% FBS in 96-well plates. After overnight incubation, different concentrations of free doxorubicin (Dox), Dox-loaded nanosphere (Dox-Nanosphere), and Dox-loaded aptamer-conjugated nanosphere (Dox-Apt-Nanosphere) were added to the fresh medium and incubated for 24 h in a humidified atmosphere with 5% CO_2_ at 37 °C. After incubation, 10 µL of cell counting kit-8 (CCK-8) solution (Dojin Laboratory, Kumamoto, Japan) was added and incubated for 3 h. Absorbance was measured at 450 nm using an Epoch-2 microplate spectrophotometer (BioTek Instruments).

### 2.8. Flow Cytometric Analysis

Flow cytometric analysis was performed to evaluate the selective binding ability of AS1411 aptamer-conjugated nanospheres to target cells. The cells (1 × 10^5^ cells per well) were seeded in 500 µL DMEM medium supplemented with 10% FBS in 24-well plates. After overnight incubation and a brief wash with 500 µL of PBS, the cells were incubated with 500 nM of FAM-labeled nanospheres and aptamer-conjugated nanospheres (Apt-Nanosphere) in 200 µL of serum-free DMEM medium for 24 h. After incubation, the cells were washed twice with PBS and resuspended in 500 µL of PBS prior to flow cytometric analysis using FACS Calibur flow cytometry (Becton Dickinson, CA, USA). Data analysis was performed using FlowJo 10.8.1 software.

### 2.9. Confocal Laser-Scanning Microscopy (CLSM)

The cells were plated in 24-well culture plates (1 × 10^5^ cells per well) at 37 °C overnight. After incubation, FAM-labeled nanospheres and Apt-Nanospheres (100 nM equivalents) were added and incubated for 24 h at 37 °C to assess selective cellular internalization. In order to compare the cellular uptake of the nanospheres, Free Dox, Dox-Nanosphere, and Dox-Apt-Nanosphere (1 µM Dox equivalent) were inoculated into the media and incubated for 4 h and 24 h. The cells were then washed with PBS and fixed with 4% paraformaldehyde. The cell nuclei were stained with 4′,6-diamidino-2-phenylindole (DAPI; Thermo Scientific, Rockford, IL, USA). Confocal imaging was performed using a Zeiss LSM710 confocal laser-scanning microscope (Carl Zeiss). The summed fluorescence intensity values were calculated using the ZEN lite 3.6 software (Carl Zeiss).

### 2.10. Three-Dimensional Tumor Spheroid Formation

The U87 cells were seeded at 6 × 10^5^ cells per well in a Stem FIT 3D cell culture dish (C100600, MicroFIT, Inc., Hanam-si, Republic of Korea). The cells were incubated in 500 µL DMEM medium supplemented with 10% FBS. After overnight incubation, 200 µL of PBS, Apt-Nanosphere, Free Dox, and Dox-Apt-Nanosphere (10 µM Dox equivalent) were added to each well, and incubation was continued for 72 h in a humidified atmosphere with 5% CO_2_ at 37 °C. After 72 h, the radius of the 3D tumor spheroids was estimated using an inverted microscope (Olympus CKX53, Tokyo, Japan) at 4 × magnification; the spheroid areas were measured using iSolution Lite 10.0 software (IMT i-solution, Burnarby, Canada). Statistical analysis was performed using one-way ANOVA with post hoc Tukey′s HSD.

### 2.11. In Vitro BTB Penetration Assays

The in vitro BTB model, established by co-culturing hCMEC/D3 and U87 cells, was used as previously described [68,69]. Briefly, the U87 cells were seeded into the basolateral chambers of the transwell plates at a density of 1.12 × 10^6^ per well, and, after 3 days, hCMEC/D3 were seeded onto the upper inserts of the transwell plates at a density of 1.12 × 10^5^ per well. The co-cultured cells were incubated for 5 days, and the culture medium (0.5 mL) was changed once. After incubation, the transendothelial electrical resistance (TEER) of the hCMEC/D3 cell monolayer was measured using an EVOM3 device (WPI, Sarasota, FL, USA). After the removal of the entire medium from both sides, 0.1 mM Free Dox and Dox-Apt-Nanosphere in 0.5 mL fresh, complete cell culture medium was added to the apical chamber and incubated with the cell monolayer for 30, 60, 120, and 300 min. At each time point, the fluorescence of Dox (Ex/Em: 470/560) from the basolateral side of the medium containing the different samples was determined using a BioTek Cytation 5 fluorescence microplate reader. The transport efficiency (TE) of each sample across the BTB model was calculated as TE = ($F_{time}$ − $F_{media}$)/($F_{total}$ − $F_{media}$), where $F_{time}$, $F_{total}$, and $F_{media}$ represent the fluorescence signals from the basolateral side of the media, containing the different samples at each time point, the basolateral side, containing the full amount of each sample, and the blank control, respectively. In addition, the transport ratio of each sample, normalized to each transwell membrane TE only, was calculated as transport ratio (TR) = ${(TE}_{BTB})/({TE}_{membrane})$.

### 2.12. In Vivo Anti-Tumor Experiments

The Institutional Animal Care and Use Committee in Seoul National University Bundang Hospital (SNUBH IACUC, No. BA-2110-329-007-06) approved all experiments. In order to generate a GBM xenograft model, 6-week-old male Balb/c nude mice were injected subcutaneously into the right thigh with U87 cells at a dose of 1 × 10^6^ cells in 100 µL PBS; the mice were randomly divided into four groups: PBS, Free Dox, Apt-Nanosphere, and Dox-Apt-Nanosphere when the tumors in tumor-bearing mice reached approximately 2 mm in diameter (*n* = 3). The samples in a volume of 100 µL per group were administered by tail vein injection with an equivalent concentration of Dox at 2 mg/kg, administered intravenously every 3 days for a total of five injections. Fluorescence images (660/710 nm) of the mice were acquired using an IVIS Caliper Lumina imaging system (Perkin Elmer, Shelton, CT, USA) to monitor the Cy5.5-labeled nanosphere. The tumor-bearing mice were sacrificed on day 5 after full treatment, and the tumor tissues were resected, fixed in formalin, and embedded in paraffin. The tumor tissues were sectioned (4 μm in thickness) and stained with hematoxylin and eosin (H&E) and DAPI using ProLong gold mounting medium with DAPI (Invitrogen). The tissue sections were examined using a Zeiss LSM710, a confocal laser-scanning microscope (Carl Zeiss). Tumor volume was estimated using calipers by entering a value of V = 0.5 × L × W^2^, where V represents tumor volume, L represents tumor length, and W represents tumor width.

### 2.13. Statistical Analysis

The results are expressed as the mean and standard error of the mean. Significance (** *p* < 0.01 and * *p* < 0.05) was assessed using the non-parametric Mann–Whitney test using GraphPad Prism 9.0 (San Diego, CA, USA).

## 3. Results

### 3.1. Generation and Characterization of AS1411 Aptamer-Conjugated Nanospheres

The nanosphere synthesis process was designed as a two-step self-assembly process in which the ssDNA oligonucleotides of the semi-complementary sequences were hybridized to form a dsDNA chain, and then the self-assembled structure was extended using a system in which biotin linked to ssDNA binds to streptavidin. The ssDNA oligonucleotide sequences [64] and the process for synthesizing a stabilized dsDNA-streptavidin hybrid nanosphere were obtained in previous studies [35,70,71]. In addition, a dsDNA-streptavidin hybrid nanosphere that selectively targets GBM cells was developed based on the AS1411 aptamer-targeting NCL proteins.

**Figure 1:**
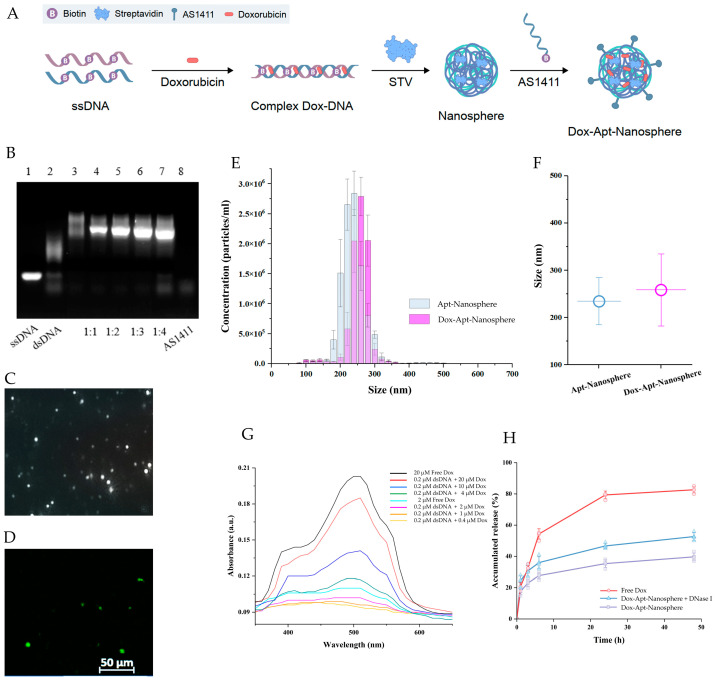
Generation of AS1411 aptamer-conjugated nanospheres. (**A**) Schematic of the Dox-Apt-Nanosphere self-assembling to transport drugs targeting tumor cells for anti-tumor effects. The biopolymer intercalates Dox into the dsDNA structure and induces biotin–streptavidin molecular binding. (**B**) Determination of the optimization of the aptamer ratios that combine with the nanospheres. A total of 2 µL of the samples were loaded on a 3% agarose gel. Lanes 1 to 3 correspond to an ssDNA (100 µM), dsDNA, and nanospheres, respectively. Lanes 4 to 7 correspond to different ratios of aptamers-to-nanospheres at 1:1, 1:2, 1:3, and 1:4, respectively. The samples in each lane were prepared by mixing a material at a concentration of 20 µM, respectively. Lane 8 corresponds to AS1411 (5 µM). (**C**) Images of Dox-Apt-Nanosphere were identified using the NanoSight NS300 system, and (**D**) FAM-labeled Dox-Apt-Nanosphere was identified using confocal microscopy. (**E**,**F**) The size distribution of Apt-Nanospheres and Dox-Apt-Nanospheres was analyzed using the NanoSight NS300 system. (**G**) The fluorescence spectra of Dox at various concentration ratios (dsDNA:Dox = 1:2, 1:5, 1:10, 1:20, 1:50, and 1:100). (**H**) The fluorescence signals of Dox-accumulated release in Dox-Apt-Nanospheres under treatment with DNase I enzyme (1 U/mL).

In order to synthesize the structure of AS1411 aptamer-conjugated nanospheres, it was necessary to verify the optimization of the ratio of AS1411 aptamers for binding to nanospheres. When the nanospheres and AS1411 aptamers were combined in a ratio of three times or more, the residual amount of ssDNA and AS1411 was hardly observed (Figure 1B). In this study, the self-assembly process was performed using the ratio at which no dimers were formed, and the ratio was as follows: [ssDNA(40 bp)]:[biotin-connected to [ssDNA(40 bp)]:[streptavidin]:[biotin-connected to 1411 aptamer(26 bp)] = 1:1:1:1:3.

With the addition of biotinylated AS1411 aptamers, it is assumed that ssDNA residues and dimers can be generated due to tertiary structure loss or non-specific binding during recombination with complementary strands [72,73]. The AS1411 residue, which is no longer bound to the nanospheres, may hinder the effectiveness of the synthesized Dox-Apt-Nanosphere if it binds to GBM cells. Therefore, the AS1411 aptamer was not synthesized in more than a three-fold ratio.

The size distribution of the AS1411 aptamer-conjugated nanospheres was identified using NanoSight NS300 (Figure 1C). Confocal microscopy was used to confirm the FAM dye of Dox-Apt nanosphere labeling (Figure 1D). The size distribution of the AS1411 aptamer-conjugated nanospheres was measured by injecting them into the NanoSight NS300 using a syringe (Figure 1E,F). Figure 1E shows the measurement of the AS1411 aptamer-conjugated dsDNA–streptavidin hybrid nanospheres at a concentration of 1.48 × 10^8^ particles per 1 mL, with an average size of 234.6 ± 44.2 nm (mean ± SD). The size of the AS1411 aptamer-conjugated dsDNA–streptavidin hybrid nanospheres with Dox was measured at a concentration of 1.74 × 10^8^ particles per 1 mL, with an average size of 260.4 ± 84.9 nm (mean ± SD). The addition of Dox increased the size by approximately 25 nm (Figure 1F).

Dox, which is used as an anti-cancer drug, can be intercalated into the “GC” or “CG” sequence of DNA due to aromatic rings [74,75]. In order to quantitatively predict the drug-bearing amount of Dox, the fluorescence spectra of Dox was measured at various concentration ratios (dsDNA:Dox = 1:2, 1:5, 1:10, 1:20, 1:50, and 1:100) (Figure 1G). The absorbance level was found to increase when the amount of Dox exceeded that of dsDNA at a ratio of 1:10 or higher. This finding is consistent with previous studies showing that 94% of the fluorescent signal of Free Dox is quenched in PBS at a dsDNA:Dox concentration ratio of 1:10, with a significant difference in absorbance observed at concentrations above 10-fold [35,76]. Therefore, the concentration ratio of dsDNA to Dox was treated as 1:10 in order to quantitatively predict the Dox released from nanosphere-encapsulated drugs.

In order to confirm the stability of the AS1411 aptamer-conjugated nanosphere structure, which is affected by nuclease degradation, we treated the nanocarriers with a level of DNase I of more than twice that found in human serum [66,67]. We confirmed that when DNase I was applied, the drug release was within 20% of that observed in the untreated Dox-Apt-Nanosphere. This confirmed that the effect of DNase I was relatively stable (Figure 1H).

### 3.2. Ability to Selectively Bind Glioblastoma Cells to AS1411 Aptamer-Conjugated Nanospheres

Multifunctional NCL proteins, which promote tumor initiation and progression, are highly expressed in various tumors. AS1411 is a 26-mer G-rich DNA aptamer, used as a targeting agent to deliver small molecules to cancer cells that overexpress NCL [52,77]. In addition, NCL acts as a molecular chaperone to support AS1411 in cell entry [52] and was, therefore, used in this study for the purpose of GBM cell-specific binding and cellular internalization.

**Figure 2:**
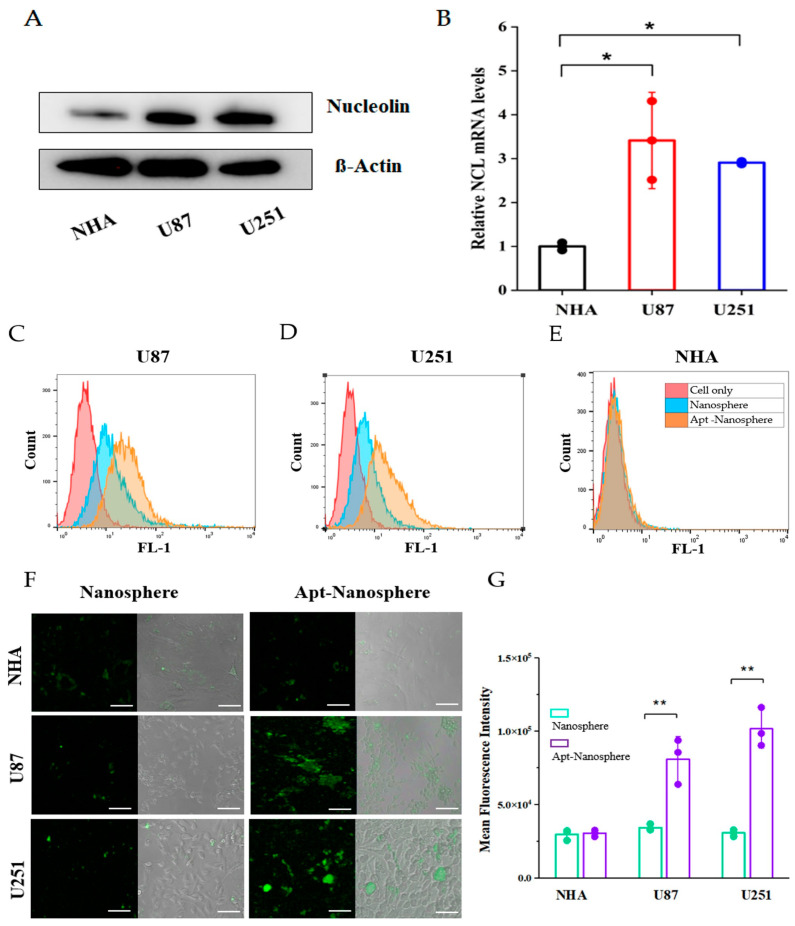
Nucleolin mRNA and protein expression analysis and flow cytometry and confocal microscopy images of non-target NHA cells and target U87 and U251 glioblastoma cells treated with nanosphere and Apt-Nanosphere. (**A**) Western blot analysis of NCL mRNA expression level of NHA, U87, and U251 cells; β-actin was used as an internal control. (**B**) Real-time RT-PCR analysis of NCL mRNA expression level of NHA, U87, and U251 cells; GAPDH was used as an internal control. Selective binding of AS1411 Apt-Nanosphere to target the U87 (**C**) and 251 glioblastoma cells (**D**) and non-target NHA cells (**E**). (**F**) A comparison of the binding affinity of the FAM-labeled nanospheres and 1411 aptamer-conjugated FAM-labeled nanospheres in the target U87 and 251 glioblastoma cells, as well as the non-target NHA cells. The scale bar for all images is 20 µm. (**G**) The mean fluorescence intensity values were calculated using the ZEN lite software. The values are expressed as mean ± SEM: ** *p* < 0.01 and * *p* < 0.05 (*n* = 6). Relative quantification was performed using the comparative Ct method (2^−ΔΔCt^). The values are expressed as mean ± SEM: ** *p* < 0.01 and * *p* < 0.05 (*n* = 4).

The expression of NCL mRNA and protein was detected in NHA cells and GBM cells (U87 and U251) using real-time RT-PCR and Western blot assays. The qRT-PCR results showed that the NCL mRNA levels were significantly higher in the GBM cells (U87 and U251) compared to the non-target NHA cells (3.41 ± 1.09 and 2.90 ± 0.03 vs. 1 ± 0.09, *p* < 0.05, Figure 2B). Similarly, in the Western blot analyses, the expression of NCL protein was significantly higher in the GBM cells (U87 and U251) than in the NHA cells (Figure 2A).

After the incubation of FAM-labeled nanospheres and FAM-labeled AS1411 aptamer-conjugated nanospheres in each cell for 24 h, the selective target affinity of the GBM cells (U87 and U251) compared to the non-target NHA cells was analyzed using flow cytometry (Figure 2). As a result, only the U87 and U251 cells (Figure 2C,D) confirmed their binding ability due to the fluorescence (FL-1) peak shifts when compared to the NHA cells (Figure 2E). The AS1411 aptamer-conjugated nanospheres selectively recognized the GBM cells.

In the confocal laser-scanning microscopy (Carl Zeiss) results, the mean fluorescence intensity (MFI) values were significantly different in the U87 and U251 cells compared to the NHA cells (*p* < 0.01), confirming selective binding between the FAM-labeled AS1411 aptamer-conjugated nanospheres and the GBM cells (Figure 2F,G).

### 3.3. Transport Efficiency and Cellular Uptake of AS1411 Aptamer-Conjugated Nanospheres with Doxorubicin

**Figure 3:**
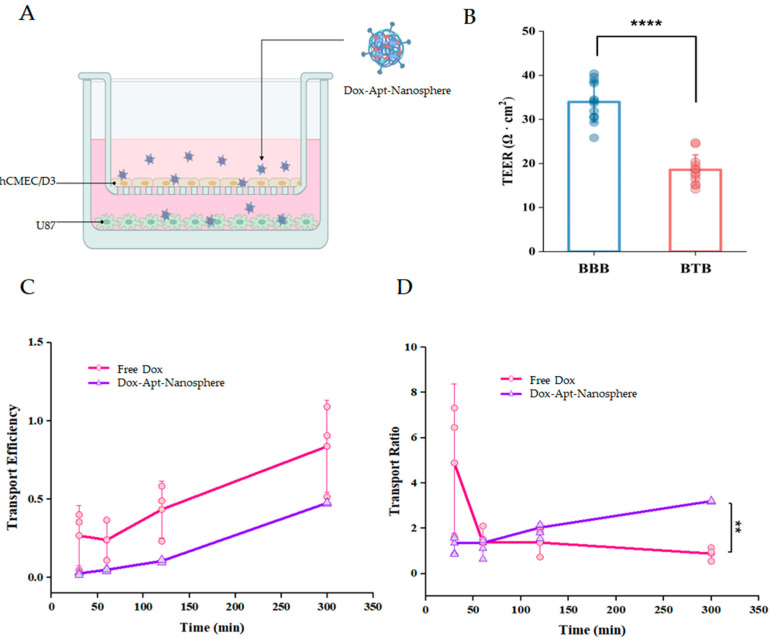
Aptamer-conjugated nanosphere penetration with doxorubicin across the in vitro BTB model. (**A**) Schematic of Dox-Apt-Nanosphere crossing the in vitro BTB model. (**B**) The TEER values of BBB and BTB are expressed as Ω ∙ cm^2^. The data represent mean ± SD (*n* = 12; each). (**C**) The transport efficiency (TE) of Free Dox and Dox-Apt-Nanosphere samples across the BTB in vitro. Transport efficiency (TE) = ($F_{time}$ − $F_{media}$)/($F_{total}$ − $F_{media}$). The data are mean ± SD (*n* = 3). (**D**) The transport ratio of Free Dox and Dox-Apt-Nanosphere was normalized to each TE in the transwell membrane. The data are mean ± SD (*n* = 3). Transport ratio (TR) = ${(TE}_{BTB})/({TE}_{membrane})$. The values are expressed as mean ± SEM: **** *p* < 0.0001; ** *p* < 0.01.

In order to compare the BTB transfer efficiency of Free Dox and Dox-Apt-Nanospheres, we attempted to establish an in vitro BTB model. In the case of GBM, the drug permeability of the BTB is high in the bulk tumor site, but there is low permeability in the periphery [2], so we established an in vitro hCMEC/D3 and U87 cell co-culture model to investigate the drug penetration efficiency and whether it could reduce drug release. In this culture system, the U87 cells stimulated a monolayer of hCMECs seeded in an insert chamber to confer an angiogenic phenotype that could mimic the BTB in vivo (Figure 3A). The measurement period for analysis was limited to 5 h prior to the cellular uptake of Free Dox. In order to assess the integrity of the BTBs, we measured the TEER after 5 days of co-culture and found that the TEER of the BTBs (18.56 ± 3.39) was significantly reduced compared to small BBB monolayers (33.95 ± 4.57) (Figure 3B). The study showed that the transport efficiency of Free Dox was consistently found to be higher than that of the aptamer-conjugated nanospheres, with a two-fold difference in efficiency across the BTB (Figure 3C). However, comparing the exact transport efficiency between the two substances is difficult because the absorbance intensity of Dox-Apt-Nanospheres is lower than that of Free Dox at the same Dox concentration. As a result, the analysis was based on the transport ratio between the membrane control group and BTB for each substance. Free Dox had a transport ratio more than three times higher than Dox-Apt-Nanospheres within 30 min. However, after 1 h, the transport ratio of Free Dox decreased, while the transport ratio of Dox-Apt-Nanospheres increased continuously. After 2 h, the transport ratio of Dox-Apt-Nanospheres was significantly higher than that of Free Dox (*p* < 0.01) (Figure 3D). It was suggested that more and more AS1411 aptamer-conjugated nanospheres were captured by NCL on the cell membrane and gradually migrated to the basolateral surface of the cell over time after penetrating the BTB.

**Figure 4:**
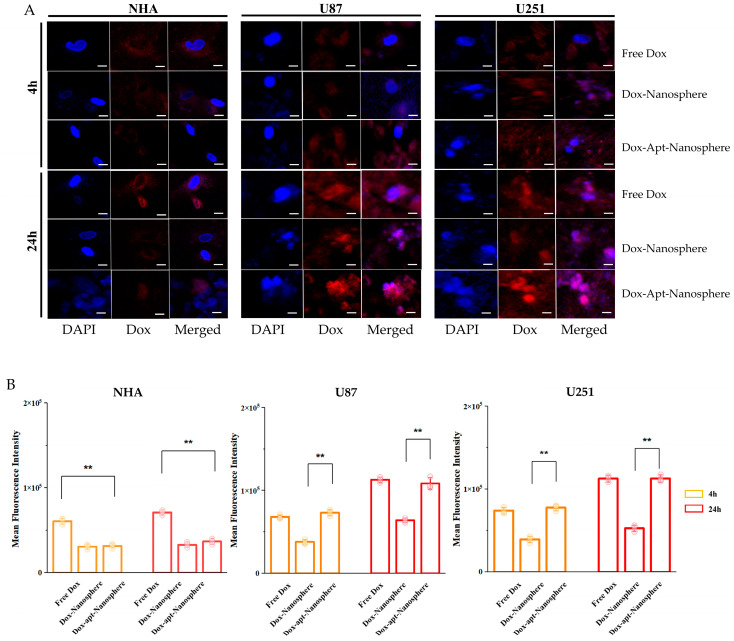
Confocal microscopy images of the cellular uptake of doxorubicin by non-target NHA cells and target U87 and U251 glioblastoma cells. (**A**) A comparison of the cellular uptake of Free Dox, Dox-Nanosphere, and Dox-AS1411 Apt-Nanosphere (1 µM Dox equivalent), which were inoculated into the media and incubated for 4 h and 24 h. The cell nuclei were stained blue with DAPI. The scale bar for all images is 10 µm. (**B**) The mean fluorescence intensity values were calculated using the ZEN lite software. The values are expressed as mean ± SEM: ** *p* < 0.01 (*n* = 6).

In order to evaluate whether AS1411 aptamer-conjugated nanospheres could selectively deliver Dox to target GBM, Free Dox, Dox-Nanospheres, and Dox-Apt-Nanospheres were inoculated into U87 or U251 target cells or the NHA non-target cells and evaluated with confocal laser-scanning microscopy (Carl Zeiss). As shown in Figure 4A,B, drug accumulation by Free Dox was observed in both the GBM (U87 and U251) and NHA cell types, and an increase in the Dox fluorescence intensity was observed at 24 h rather than 4 h. Dox-Apt-Nanosphere nanospheres were observed at a level equivalent to the fluorescence intensity of Free Dox and showed Dox accumulation over time in the U87 and U251 cells. In the GBM cells, Dox-Nanosphere nanospheres had a significantly lower cell accumulation of Dox than Dox-Apt-Nanospheres (*p* < 0.01). In the NHA cells, Dox-Apt-Nanosphere and Dox-Nanosphere nanospheres showed no increase in Dox accumulation over time, and the Dox fluorescence intensities of Free Dox and Dox-Apt-Nanospheres showed a significant difference (*p* < 0.01). The AS1411 aptamer-conjugated nanospheres with AS1411–NCL interactions in GBM cells with high NCL expression clearly showed a selective drug delivery ability similar to Free Dox.

### 3.4. Cytotoxicity of AS1411 Aptamer-Conjugated Nanospheres with Doxorubicin

**Figure 5:**
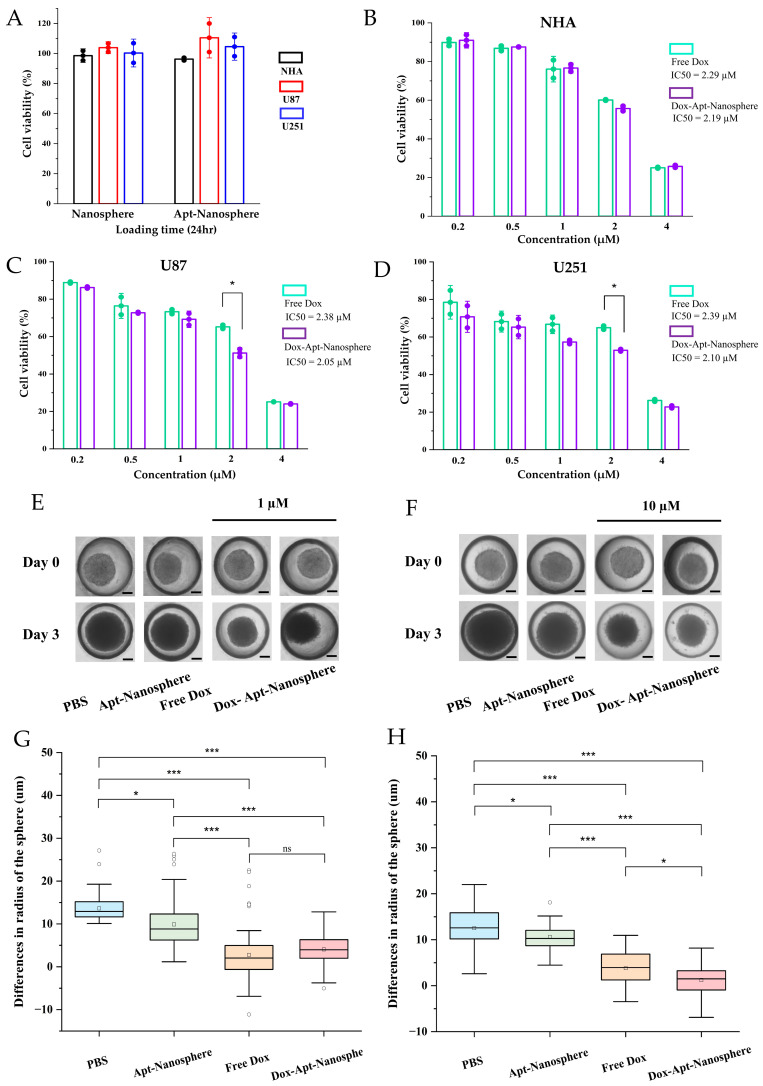
In vitro selective cytotoxicity of Dox-loaded AS1411 Apt-Nanosphere and the evaluation of the differences in the radii of 3D tumor spheroids after treatment with PBS, Apt-Nanosphere, Free Dox, and Dox-Apt-Nanosphere. (**A**) The cell viability of nanospheres without Dox against NHA, U87, and U251 cells was determined using CCK-8 assay. NCL-negative NHA (**B**) cells and positive U87 (**C**) and U251 (**D**) glioblastoma cells were incubated with Free Dox and Dox-AS1411 Apt-Nanosphere for 24 h. The treatment concentrations of Dox were 0.2, 0.5, 1, 2, and 4 µM, respectively. The values are expressed as mean ± SEM: * *p* < 0.05 (*n* = 5). (**E**) Optical microscopy images of U87 spheroids were captured after treatment with PBS (*n* = 78), Apt-Nanosphere (*n* = 78), Free Dox (*n* = 83), and Dox-Apt-Nanosphere (*n* = 84) for 0 and 3 days. The scale bar for all images is 20 µm. (**F**) Optical microscopy images of U87 spheroids were captured after treatment with PBS (*n* = 76), Apt-Nanosphere (*n* = 49), Free Dox (*n* = 41), and Dox-Apt-Nanosphere (*n* = 58) for 0 and 3 days. The scale bar for all images is 20 µm. (**G**) The differences in the radii of the U87 spheroids were calculated after 3 days of treatment with 1 µM Dox. (**H**) The differences in the radii of the U87 spheroids were calculated after 3 days of treatment with 10 µM Dox. The data were analyzed using one-way ANOVA with Tukey HSD post hoc testing. The values are expressed as mean ± SEM: *** *p* < 0.001; * *p* < 0.05; *ns,* no significant difference.

CCK-8 cytotoxicity assays were performed to determine whether Dox-loaded AS1411 aptamer-conjugated nanospheres could target GBM cells to promote drug-induced killing in target cells (Figure 5).

First, to assess the toxicity of the nanospheres themselves, the nanospheres and aptamer-conjugated nanospheres were inoculated into U87 and U251 target cells and NHA non-target cells, respectively; no evidence of cytotoxicity was observed in the nanospheres without Dox loading (Figure 5A). Free Dox showed dose-dependent cytotoxicity against both NHA and GBM U87 and U251 target cells. However, the Dox-Apt-Nanosphere exhibited drug-induced cell death effects that were specific to GBM U87 (Figure 5C) and U251 (Figure 5D) cells at a concentration of around IC50. In contrast, no significant difference was observed in the NHA cells (Figure 5B). The IC50 values for inhibiting the growth of the U87 and U251 GBM cells were 2.38 μM and 2.39 μM for Free Dox and 2.05 μM and 2.10 μM for the Dox-Apt-Nanosphere, respectively. Notably, the Dox-Apt-Nanosphere exhibited lower IC50 values than Free Dox for both cell lines. These comparisons of targeted and non-targeted cells demonstrated that selective cell killing can be achieved by the precise delivery of anti-cancer drugs using AS1411 aptamer-conjugated nanospheres with Dox.

### 3.5. Anti-Tumor Effect of AS1411 Aptamer-Conjugated Nanospheres with Doxorubicin

Drug delivery and efficacy were evaluated in the 3D models of U87 spheroids, which more closely resemble in vivo tumor characteristics than 2D models (Figure 5). In the 2D model, the Apt-Nanosphere alone did not inhibit tumor growth, but in the 3D model, the Apt-Nanosphere inhibited cell growth significantly more than PBS (*p* < 0.05), and the AS1411 aptamer itself had some effect on growth inhibition by binding to cells. Both the Free Dox and Dox-Apt-Nanosphere showed a significant inhibition of tumorigenesis compared to PBS and Apt-Nanosphere at 1 µM (*p* < 0.001; Figure 5E,G), a concentration similar to that in the 2D model; however, at 10 µM, which is 10 times higher than in the 2D model, Dox-Apt-Nanosphere showed significantly higher tumorigenesis inhibition efficacy than Free Dox (*p* < 0.05; Figure 5F,H). We confirmed that the 3D model requires a higher concentration of drug to be induced than the 2D model [78] and that, at the same concentration of Dox, Dox-Apt-Nanosphere would adhere to the GBM cells and inhibit the cell growth with a higher drug delivery efficiency than Free Dox.

**Figure 6:**
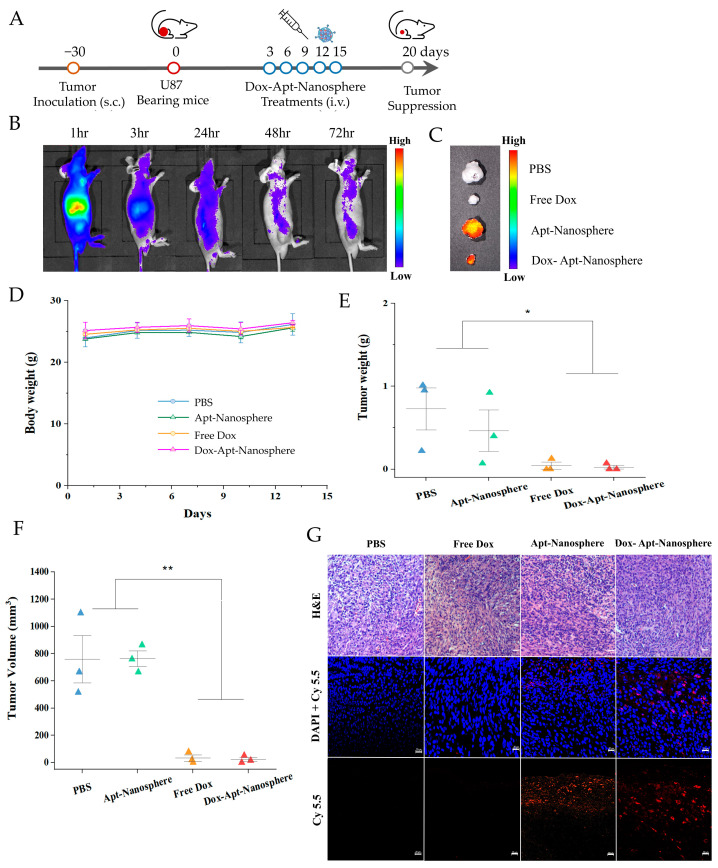
Anti-tumor effects of PBS, Apt-Nanosphere, Free Dox, and Dox-Apt-Nanosphere in a xenograft tumor model. (**A**) Schematic of Dox-Apt-Nanosphere treatment to suppress tumor growth. (**B**) In vivo biodistribution analysis using IVIS imaging of U87 cell tumor-bearing BALB/c mice after intravenous injection of Cy5.5-labeled Apt-Nanosphere. (**C**) Ex vivo imaging of tumors dissected from the mice at the end of the experiment. (**D**) Changes in body weight of the mice during treatment. (**E**,**F**) The weight and volume of the mice′s tumors were measured on day 15. The values are expressed as mean ± SEM: ** *p* < 0.01; * *p* < 0.05. (**G**) Confocal microscopy images of DAPI-stained nuclei merged the images of the DAPI and Cy-5.5 and H&E staining of tumor sections in the PBS-, Apt-Nanosphere-, Free-Dox-, and Dox-Apt-Nanosphere-treated groups. The scale bar for all images is 20 µm.

In addition, after the injection of AS1411 aptamer-conjugated nanospheres using a GBM xenograft tumor model in nude mice, the biodistribution imaging of the drug delivery to the tumor target and the tumor suppression effect was observed (Figure 6). Nanospheres were labeled with Cy5.5 and injected intravenously into the tail of U87 tumor-bearing mice. Regarding the distribution of nanospheres, the fluorescence signal was found to be the strongest, mainly in the organs, meaning the degradation of the nanospheres appeared to be active [79]; this was followed by checking the tumor target delivery image of Dox-Apt-Nanospheres for strong signals in the tumor tissue. As the fluorescence signal strength of Dox-Apt-Nanospheres distributed throughout the body of U87 tumor-bearing mice decreased after 48 h, we estimated that the drug had gradually degraded over a 72 h period (Figure 6B). In order to confirm that Dox-Apt-Nanospheres accumulated directly in the tumor tissue, the ex vivo fluorescence signal and the direct delivery to the tumor tissue were verified (Figure 6C). H&E staining of the tumor tissue showed no significant tissue necrosis and biological stability was confirmed; the fluorescence signals from the Cy5.5-labeled Dox-Apt-Nanosphere in the tissue sections demonstrated target binding and penetration into tumor tissue adjacent to blood vessels (Figure 6G). After confirming drug delivery to the tumor tissue, the anti-tumor effect of the Dox-Apt-Nanosphere was evaluated in U87 tumor-bearing mice for the PBS, Free Dox, Apt-Nanosphere, and Dox-Apt-Nanosphere treatment groups. As a result, similarly to the experimental results in the 3D spheroid model, the tendency of volume reduction in the Apt-Nanosphere group compared to the PBS group was confirmed in the U87 tumor-bearing mice model, but no statistically significant anti-tumor effect was observed (Figure 6E,F). Dox-Apt-Nanosphere also showed a tendency to have a lower volume than Free Dox, but no statistically significant difference in the anti-tumor capacity was observed. In addition, there was no significant change in body weight in either the Apt-Nanosphere- or Dox-Apt-Nanospheres-treated animals, confirming the biosafety of the drug (Figure 6D).

## 4. Discussion

GBM has a high recurrence rate and poor prognosis, and various treatment strategies are needed to prolong survival. Post-operative chemotherapy is still needed to treat GBM, and tumor-targeted drug delivery treatments using nano platform systems are still under development.

A multi-faceted understanding of the BBB/BTB is essential for CNS therapy or drug delivery strategies to the brain. As cancer progresses, it degenerates into BTB and, even within the micro-environment of the same lesion, it differs from the characteristics of the intact BBB, which may not be treatable in terms of designing drug delivery. The angiogenesis process surrounding tumors, including GBM, has irregular and inadequate underlying barriers with discontinuous epithelium. BTB forms a leaky vascular structure with 40–200 nm of fenestration [80]. This vascular structure causes the passive accumulation of drugs in tumors due to the effect of EPR in drug delivery using nanocarriers. A size of 40–200 nm may be suitable for nanoplatforms used for diagnosis and treatment and, even if the nanocarrier is smaller than 5 nm, it is easily excreted by the kidneys during the long-term circulation of the drug [81]. It is essential that BBB/BTB drug delivery is designed to accurately target and deliver drugs to cancer cells.

The nanoparticle size used in this study was confirmed to be around 100–300 nm, designed to accumulate Dox specifically in GBM cells. In addition, the drug was reasonably stable in the treatment of DNAse I, which was more than twice the human blood concentration, and the drug transport ratio into tumor cells was stable and active compared to Free Dox. NCL was overexpressed in renal vascular endothelial cells and GBM cells, and the specific targeting of cancer cells was possible through the interaction of the AS1411 aptamer with NCL. Cellular selective binding and drug uptake in cytotoxicity analysis showed a significant improvement and increase in cytotoxicity in U87 and U251 cells upon inoculation with AS1411 aptamer-conjugated nanospheres. The high rate of apoptosis of U87 and U251 cells reflected the increased toxicity for active drug delivery with the function of AS1411. Doxorubicin (DOX) is an anthracycline with potent anti-tumor activity in various cancer cells, including GBMs. Its general mechanism involves inserting base pairs into DNA strands to block replication and transcription processes, as well as inhibiting the synthesis of RNA and DNA. Additionally, it inhibits Topoisomerase II (TOP2), further preventing DNA replication, transcription, and repair [13,14]. As a result, drug delivery inhibited cell growth, with similar levels of Free Dox and Dox-Apt-Nanosphere; importantly, the NHA control cells showed less accumulation due to significant differences when drugs were delivered with AS1411 aptamer-conjugated nanospheres. The GBM xenograft tumor model also confirmed the biosafety of the tumor tissue after drug treatment and the direct delivery of Dox-Apt-Nanosphere. In the 3D spheroid model, Dox-Apt-Nanospheres significantly inhibited cell growth compared to Free Dox. Non-specific drug delivery can damage normal tissue while accumulating in the GBM tissue. Therefore, it is designed to enhance drug accumulation in tumor cells by targeting molecules that are over-expressed on the surface of tumor cells.

This study aims to apply previous research that reported nuclear deformation and cancer cell apoptosis when specifically binding to the NCL protein [77,82]. This study inhibited cancer cell proliferation using nanoparticles conjugated with the AS1411 aptamer [83]. Previous studies have shown that nanoparticles conjugated to the AS1411 aptamer are more effective in promoting intracellular drug target delivery and drug release than Free Dox [84,85]. In comparison to previous studies, a less significant difference was found in the anti-cancer effects of the drug when compared to Free Dox. However, the use of a drug carrier with high biocompatibility and self-assembly is expected to provide advantages.

The nanodrug carriers used in this study have been shown in previous studies to have the following advantages. It consists only of biomolecules containing DNA and proteins, and so high biocompatibility and biodegradability are expected. Nanopharmaceutical transporters could be induced to self-assemble biomolecules with specific molecular recognition interactions between biotin and streptavidin for the cross-linking of DNA nanostructures. In addition, the possibility of encapsulation could be explored by intercalating Dox into the DNA duplex and combining it with an aptamer that can carry cancer-cell-targeting functions.

## 5. Conclusions

In conclusion, an AS1411 aptamer-conjugated nanosphere drug delivery system was developed for the dual-target treatment of GBM. AS1411-mediated specific recognition and drug accumulation significantly improved NCL-positive U87 and U251 cell binding, and improved cytotoxicity. Improving the ability to penetrate and retain anti-tumor drugs is known to be important in clinical treatment [86]. In this study, compared to non-specific EPR-mediated delivery and passive drug delivery to tumor cells, the active targeting model using aptamer-mediated drug delivery did not obtain an absolute value in terms of improving drug penetration and retention; however, the potential of this approach was confirmed through 3D spheroid and in vivo GBM xenograft tumor model experiments. These results suggest that AS1411 aptamer-conjugated nanospheres may have excellent biocompatibility, specifically targeted drug delivery capability, and the potential to exert anti-tumor effects in the targeted therapy of GBM cells.

## Data Availability

The data presented in this study are available upon request from the corresponding author.
